# The Impact of Surgical Timing on Outcomes Following Hip Fracture Surgery in Patients Receiving Direct Oral Anticoagulants: A Systematic Review and Meta-Analysis

**DOI:** 10.7759/cureus.113458

**Published:** 2026-07-27

**Authors:** Andrea Noah Paris, Nicole Galdes, Duncan Marston, Denise Galdes, Prabu Balasubramanian

**Affiliations:** 1 Surgery, Mater Dei Hospital, Msida, MLT; 2 Internal Medicine, Mater Dei Hospital, Msida, MLT; 3 Trauma and Orthopaedics, The Royal London Hospital, London, GBR

**Keywords:** direct oral anticoagulant, doac, early surgery, hip fracture surgery, meta-analysis, systematic review, time to hip surgery

## Abstract

Hip fractures are among the most common fragility injuries in older adults. With an ageing population and increasing use of direct oral anticoagulants (DOACs), the number of anticoagulated hip fracture patients continues to rise. However, the optimal timing of surgery in this population remains controversial.

A systematic review and meta-analysis was conducted in accordance with Preferred Reporting Items for Systematic Reviews and Meta-Analyses (PRISMA) guidelines. A literature search of PubMed, Embase, and the Cochrane Library (January 2010-January 2026) identified six eligible cohort studies, comprising one prospective and five retrospective studies, comparing early (<24 hours) versus delayed (24-48 hours) hip fracture surgery in patients receiving DOAC therapy. Primary outcomes measured perioperative bleeding, assessed by change in haemoglobin concentration and postoperative transfusion requirements. Secondary outcomes included length of hospital stay, postoperative complications, 30- and 90-day all-cause mortality, and operative time.

Meta-analysis of six studies demonstrated no significant difference in postoperative transfusion requirements between early and delayed surgery (odds ratio (OR) 1.18, 95% confidence interval (CI) 0.91-1.52; I² = 12%). Meta-analysis of four studies demonstrated a small but statistically significant reduction in perioperative haemoglobin drop in patients undergoing delayed surgery (mean difference (MD) 0.29 g/dL, 95% CI 0.01-0.58; I² = 12%). Early surgery was associated with a significantly shorter hospital length of stay (MD -1.27 days, 95% CI -2.39 to -0.15; I² = 34%) and a lower risk of postoperative complications (OR 0.49, 95% CI 0.31-0.78; I² = 13%). No significant difference in 30- and 90-day mortality was observed between groups (OR 1.03, 95% CI 0.47-2.27; I² = 0%).

Early hip fracture surgery in DOAC-treated patients was not associated with increased postoperative transfusion requirements or mortality compared with delayed surgery. Although delayed surgery was associated with a statistically significant reduction in perioperative haemoglobin decline, the magnitude of this difference is unlikely to be clinically significant. Early surgery was associated with fewer postoperative complications and a shorter hospital length of stay, supporting expedited operative management in appropriately selected patients receiving DOAC therapy.

## Introduction and background

Hip fractures are one of the most common fragility injuries encountered in older adults [1]. Population ageing has contributed to a rising incidence of hip fractures, placing a considerable clinical and economic strain on healthcare resources worldwide [2]. Surgical intervention, either through osteosynthesis [3,4] or arthroplasty [5], remains the standard of care. Early surgery is widely recognised as a key determinant of outcome and has consistently been associated with lower rates of morbidity and mortality [6].

The 2021 American Academy of Orthopaedic Surgeons (AAOS) clinical practice guidelines recommend operative management of hip fractures within 24-48 hours to optimise patient outcomes [7]. Subsequently, higher-level evidence has suggested that an even shorter time to surgery may be advantageous. Welford et al., in a systematic review and meta-analysis, demonstrated that earlier surgery, performed within 24 hours of admission, was associated with lower mortality and improved clinical outcomes when compared with surgery performed after 24 hours [8]. Despite these recommendations, delays to hip fracture surgery remain common, with anticoagulant therapy representing one of the leading patient-related causes and accounting for up to 10% of surgical delays [9].

Direct oral anticoagulants (DOACs), including rivaroxaban, apixaban, edoxaban and dabigatran, are widely prescribed for stroke prevention in patients with atrial fibrillation [10] and for the treatment and secondary prevention of venous thromboembolism [11]. Compared with warfarin, DOACs offer a more favourable efficacy and safety profile, together with predictable pharmacokinetics, fixed dosing regimens, and no requirement for routine coagulation monitoring [12]. As a result, DOAC use has increased substantially, and a growing proportion of patients presenting with hip fractures are anticoagulated with these agents at the time of injury [13].

Compared to warfarin and heparin, DOACs are associated with more limited access to immediate reversal strategies, often leading clinicians to delay surgery until adequate drug clearance has occurred [14]. Sundet et al. reported a median time to surgery of 36 hours (95% confidence interval (CI) 35-38) in patients receiving DOAC therapy compared with 17 hours (95% CI 17-18) in non-anticoagulated patients [15]. In patients with normal renal and hepatic function, DOACs have an elimination half-life of approximately 10-12 hours, with clinically significant drug clearance typically achieved within 24 hours [16]. However, this timeframe may be prolonged in older adults, who frequently have impaired renal function due to chronic kidney disease or acute kidney injury at presentation [16]. Furthermore, assessment of residual anticoagulant activity is challenging, as routine coagulation tests, including the international normalised ratio (INR) and activated partial thromboplastin time (aPTT), correlate poorly with DOAC activity and therefore provide limited guidance when assessing perioperative bleeding risk [17].

In an effort to minimise unnecessary delays, the 2024 Association of Anaesthetists (AoA) guidelines recommend that patients receiving DOAC therapy may proceed to hip fracture surgery within 36 hours of the last administered dose [18]. However, this recommendation is based predominantly on pharmacokinetic principles and expert opinion, rather than high-quality clinical evidence [18]. Consequently, uncertainty remains regarding the optimal timing of surgery in patients receiving DOAC therapy, particularly when balancing the risk of perioperative bleeding against the well-established risks associated with delaying hip fracture surgery.

The aim of this systematic review and meta-analysis was to evaluate the impact of surgical timing on clinical outcomes in patients with hip fractures receiving DOAC therapy, comparing surgery performed within 24 hours of the last DOAC dose with surgery undertaken between 24 and 48 hours after the last DOAC dose.

## Review

Methods

This systematic review and meta-analysis was conducted in accordance with the Preferred Reporting Items for Systematic Reviews and Meta-Analyses (PRISMA) guidelines [19] and was prospectively registered with the International Prospective Register of Systematic Reviews (PROSPERO) (registration number: CRD420261306925) [20].

Search Strategy

A systematic literature search was conducted in February 2026 using the following electronic databases: PubMed [21], Embase [22], and the Cochrane Library [23]. Medical Subject Headings (MeSH) terms and free-text keywords were combined to optimise search sensitivity and specificity. The following search terms were used: hip fracture, hip fractures, intertrochanteric fracture, neck of femur fracture, subtrochanteric fracture, intracapsular hip fracture, extracapsular hip fracture, proximal femur fracture, direct oral anticoagulant, DOAC, novel oral anticoagulant, NOAC, rivaroxaban, apixaban, dabigatran, edoxaban, surgical timing, time to surgery, early surgery, delayed surgery, <24 hours, >24 hours, and 24-48 hours. Boolean operators (AND, OR), together with the appropriate use of parentheses and quotation marks, were applied to combine search terms and improve search accuracy.

Eligibility Criteria

Eligibility criteria followed the population-intervention-comparator-outcome-study design (PICOS) framework (Table 1). Eligible studies were randomised controlled trials, prospective or retrospective cohort studies, and case-control studies evaluating adults (≥18 years) with hip fractures receiving DOAC therapy. Studies were required to compare surgery performed within 24 hours of the last DOAC dose, with surgery undertaken between 24 and 48 hours after the last DOAC dose, and to report at least one predefined primary outcome. Studies were excluded if they included patients receiving anticoagulants other than DOACs, unless outcomes specific to DOAC-treated patients could be analysed separately; used alternative definitions of early or delayed surgery; were non-comparative studies, case reports, reviews, editorials, letters, conference abstracts, or expert opinion articles; or were published before January 2010, in a language other than English, or without full-text availability.

**Table 1:** PICOS framework-based study inclusion and exclusion criteria PICOS: population-intervention-comparator-outcome-study design; DOAC: direct oral anticoagulant

PICOS	Inclusion Criteria	Exclusion Criteria
Population	Adults (≥18 years) with neck of femur, intertrochanteric, or subtrochanteric hip fractures receiving DOAC therapy (apixaban, rivaroxaban, dabigatran, or edoxaban).	Patients receiving anticoagulants other than DOACs (eg., warfarin, heparin, or antiplatelet-only therapy), unless outcomes specific to DOAC-treated patients could be analysed separately. Studies involving concomitant anticoagulant therapy where the effect of DOACs could not be distinguished.
Intervention	Early surgery performed within 24 hours of the last DOAC dose.	Studies defining early surgery using time thresholds other than <24 hours after the last DOAC dose.
Comparator	Delayed surgery performed 24-48 hours after the last DOAC dose.	Studies defining delayed surgery using time thresholds other than 24-48 hours after the last DOAC dose.
Outcomes	Studies reporting at least one predefined outcome. Primary outcomes: perioperative bleeding defined by a change in haemoglobin concentration or blood transfusion requirements. Secondary outcomes: length of hospital stay, postoperative complications, 30-day mortality, 90-day mortality, and operative time.	Studies that did not report any predefined primary or secondary outcome.
Study design	Randomised controlled trials, prospective or retrospective cohort studies, and case-control studies published in peer-reviewed journals.	Non-comparative studies, case reports, narrative reviews, systematic reviews, editorials, letters, conference abstracts, and expert opinion articles.
Publication	Full-text articles published in English.	Studies published before January 2010, non-English-language publications, or articles for which full text was unavailable.

Study Selection

The search was limited to studies published from January 2010 onwards. All identified records were uploaded to Rayyan.ai (Rayyan Systems Inc., Cambridge, USA) [24]. Duplicate records were identified using the platform's built-in duplicate detection tool and subsequently verified manually before removal. Title and abstract screening was performed independently by two reviewers. Any disagreements were resolved through discussion with a third reviewer. Full-text articles were then retrieved and independently assessed for eligibility by two reviewers, with studies meeting the predefined eligibility criteria included in the review.

Data Extraction

Data were extracted manually using a standardised Excel data extraction form (Microsoft Corp., Redmond, USA). The following variables were collected from each included study: author, year of publication, study design, total number of participants, number of participants within the <24-hour and 24-48-hour groups, type of surgical procedure performed, perioperative change in haemoglobin concentration, number of patients requiring blood transfusion, length of hospital stay, 30-day and 90-day all-cause mortality, postoperative complications, and operative time.

Data Analysis

Continuous outcomes were analysed using mean differences (MDs) with corresponding 95% CIs, while dichotomous outcomes were analysed using odds ratios (ORs) with 95% CIs. Meta-analyses were performed for all primary and secondary outcomes reported by at least three studies using ReviewManager (RevMan) Web (The Cochrane Collaboration, London, UK) [25]. Statistical heterogeneity was assessed using the I² statistic, with values of 25%, 50%, and 75% representing low, moderate, and high heterogeneity, respectively. Statistical significance was defined at a p-value < 0.05. Forest plots were generated using RevMan Web.

Risk of Bias

As all included studies were observational, methodological quality and risk of bias were assessed using the Risk of Bias in Non-randomised Studies of Interventions (ROBINS-I) tool [26]. Domain-level and overall risk-of-bias assessments were visualised using the Risk-of-Bias Visualisation (ROBVIS) tool [27]. In accordance with Cochrane recommendations, publication bias was not analysed because fewer than ten studies were available for each meta-analysis.

Results

**Figure 1:**
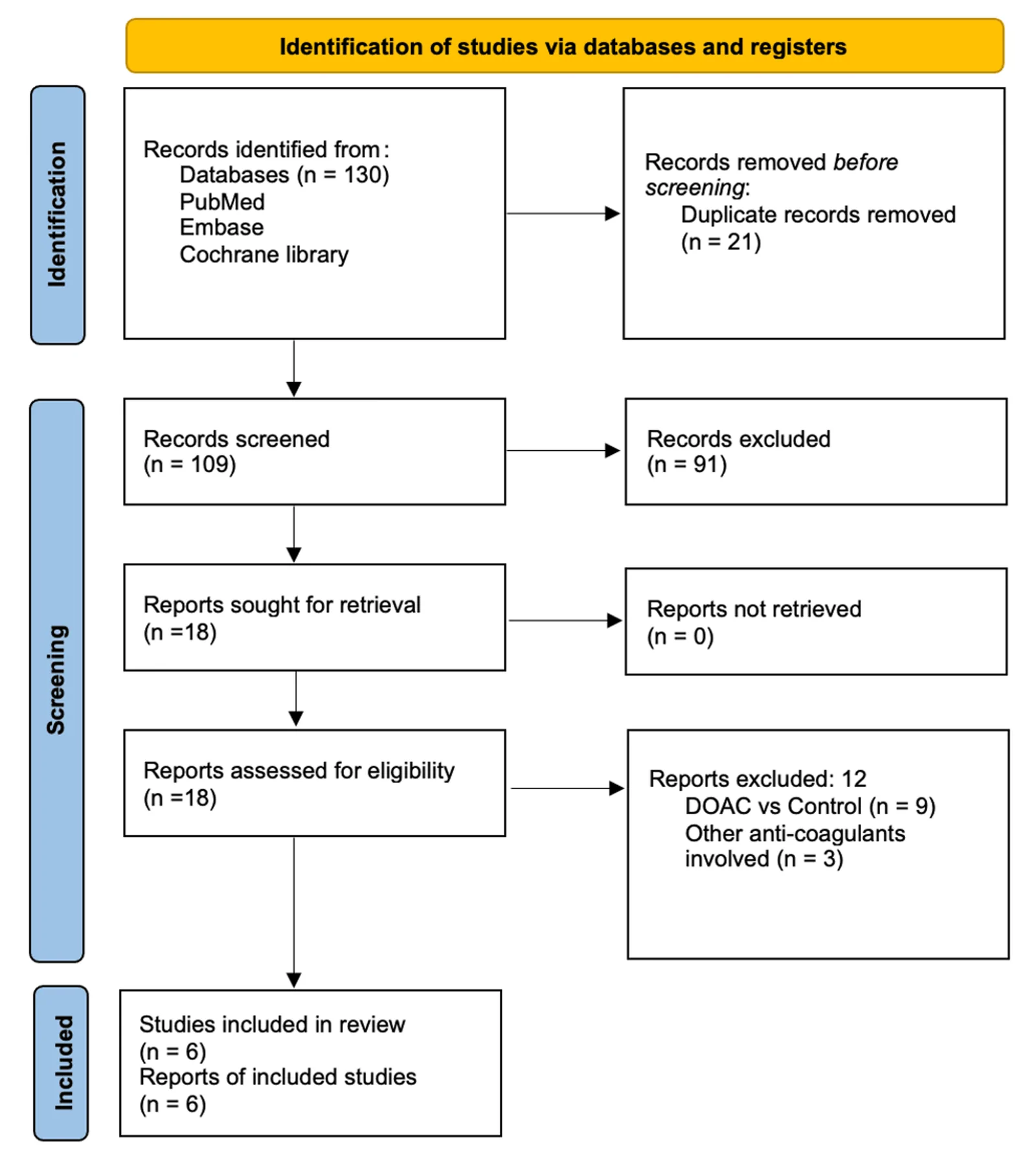
PRISMA flow diagram PRISMA: Preferred Reporting Items for Systematic Reviews and Meta-Analyses; DOAC: direct oral anticoagulant

The database search identified 130 records, of which 21 duplicates were removed. The remaining 109 records underwent title and abstract screening, following which 18 articles were considered potentially eligible and retrieved for full-text assessment. After application of the predefined eligibility criteria, 12 studies were excluded, leaving six studies for inclusion in the systematic review and meta-analysis. The study selection process is summarised in the PRISMA flow diagram (Figure 1).

Study Characteristics

A total of 1,575 patients were included across the six studies [28-33], of whom 728 underwent surgery within 24 hours and 847 underwent surgery between 24 and 48 hours of the last DOAC dose.

Weihs et al. [29] was the only prospective cohort study included, whereas the remaining five studies employed retrospective cohort designs [28,30-33]. Schiepers et al. [33] was the only multicentre study, while the remaining studies [28-32] were conducted at single institutions.

Considerable clinical heterogeneity existed with respect to fracture pattern and surgical management. Chen et al. exclusively included patients with intracapsular hip fractures treated with hemiarthroplasty or total hip arthroplasty [28]. In contrast, Weihs et al. evaluated only extracapsular hip fractures managed with cephalomedullary nailing [29]. The remaining four studies included both intra- and extracapsular fractures, with patients undergoing either osteosynthesis or arthroplasty according to fracture configuration and local institutional practice [30-33].

Primary outcome reporting demonstrated methodological heterogeneity. Four studies quantified perioperative blood loss as the change in haemoglobin concentration (g/dL) [28,30-32], whereas Weihs et al. [29] reported estimated blood loss (mL) and Schiepers et al. [33] reported the median reduction in haemoglobin concentration (mmol/L). Postoperative transfusion requirements were reported in all six studies [28-33].

Secondary outcomes included 30-day mortality [28,32,33], 90-day mortality [28,30,32], length of hospital stay [28,30,31], postoperative complications [29-31], and operative time [28,33]. Reporting of these outcomes was heterogeneous across the included studies.

Chen et al. [28] and Krespi et al. [32] also reported outcomes for patients undergoing surgery more than 48 hours after the last DOAC dose. These cohorts were excluded from the quantitative synthesis to maintain consistency with the predefined comparison of early (<24 hours) versus delayed (24-48 hours) surgery.

A summary of the study characteristics and outcomes reported by each study is presented in Table 2.

**Table 2:** Study characteristics and reported outcomes NR: not recorded; HA: hemi-arthroplasty; THA: total hip arthroplasty; SHS: sliding hip screw; CMN: cephalomedullary nail; Hb: haemoglobin; LOS: length of stay

Author and Year	Country	Study Design	Total Sample Size	Number of Individuals Operated <24 Hours	Number of Individuals Operated in 24-48 Hours	Type of Surgery Performed	Outcomes Reported
Chen et al., 2025 [28]	United States of America	Single-centre retrospective cohort	128	39	89	THA HA	Hb change g/dL, number of postoperative transfusions, 30-day mortality, 90-day mortality, mean LOS, operation time
Weihs et al., 2025 [29]	Austria	Single-centre prospective cohort	138	69	69	CMN	Median blood loss in mL, number of postoperative transfusions, complications
Wang et al., 2025 [30]	United States of America	Single-centre retrospective cohort	250	67	183	HA SHS CMN Cannulated screws	Hb change g/dL, number of postoperative transfusions, 90-day mortality, mean LOS complications
Devlieger et al., 2024 [31]	Switzerland	Single-centre retrospective cohort	59	17	42	NR	Hb change g/dL, number of postoperative transfusions, mean LOS, complications
Krespi et al., 2022 [32]	Israel	Single-centre retrospective cohort	125	32	93	HA SHS CMN	Hb change g/dL, number of postoperative transfusions, 30-day mortality, 90-day mortality
Schiepers et al., 2026 [33]	United States of America	Multicentre retrospective cohort study	875	504	371	THA HA SHS CMN	Median Hb decrease mmol/L, number of postoperative transfusions, median operation time, median LOS, 30-day mortality

Primary Outcomes

**Figure 2:**
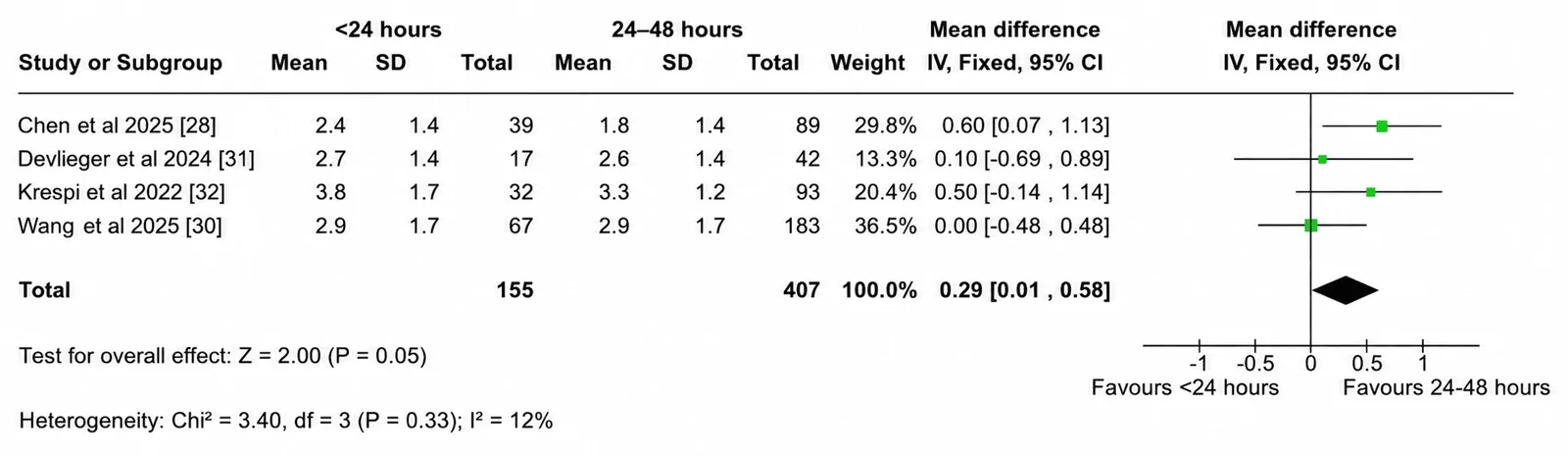
Forest plot comparing perioperative change in haemoglobin concentration between surgery performed within 24 hours and 24-48 hours after the last DOAC dose DOAC: direct oral anticoagulant; IV: inverse variance; CI: confidence interval; SD: standard deviation; df: degrees of freedom [28,30-32]

Blood loss: Four studies [28,30-32] reported perioperative changes in haemoglobin concentration and were included in the meta-analysis. A statistically significant difference was observed between the two groups, with patients undergoing surgery between 24 and 48 hours experiencing a smaller reduction in haemoglobin concentration than those undergoing surgery within 24 hours. The pooled analysis demonstrated an MD of 0.29 g/dL in favour of the 24-48-hour group (95% CI 0.01-0.58; p = 0.05) (Figure 2). Low heterogeneity was observed across the included studies (I² = 12%).

Weihs et al. [29] reported perioperative blood loss as volume (mL) rather than change in haemoglobin concentration and was therefore not included in the meta-analysis. Median blood loss was 1,107.3 mL (interquartile range (IQR) 745.6-1,120.8) in the <24 hours group and 1,129.7 mL (IQR 928.5-1,250.0) in the 24-48 hours group, with no statistically significant difference observed between groups (p = 0.824).

Schiepers et al. [33] reported blood loss as the median reduction in haemoglobin concentration (mmol/L). Patients undergoing surgery within 24 hours experienced a significantly smaller decline in haemoglobin levels compared with those undergoing surgery between 24 and 48 hours. The median haemoglobin decrease was 0.6 mmol/L (IQR 0.0-1.4) in the early surgery group and 0.9 mmol/L (IQR 0.4-1.5) in the delayed surgery group, corresponding to a median difference of 0.3 mmol/L (95% CI 0.2-0.4; p < 0.001).

**Figure 3:**
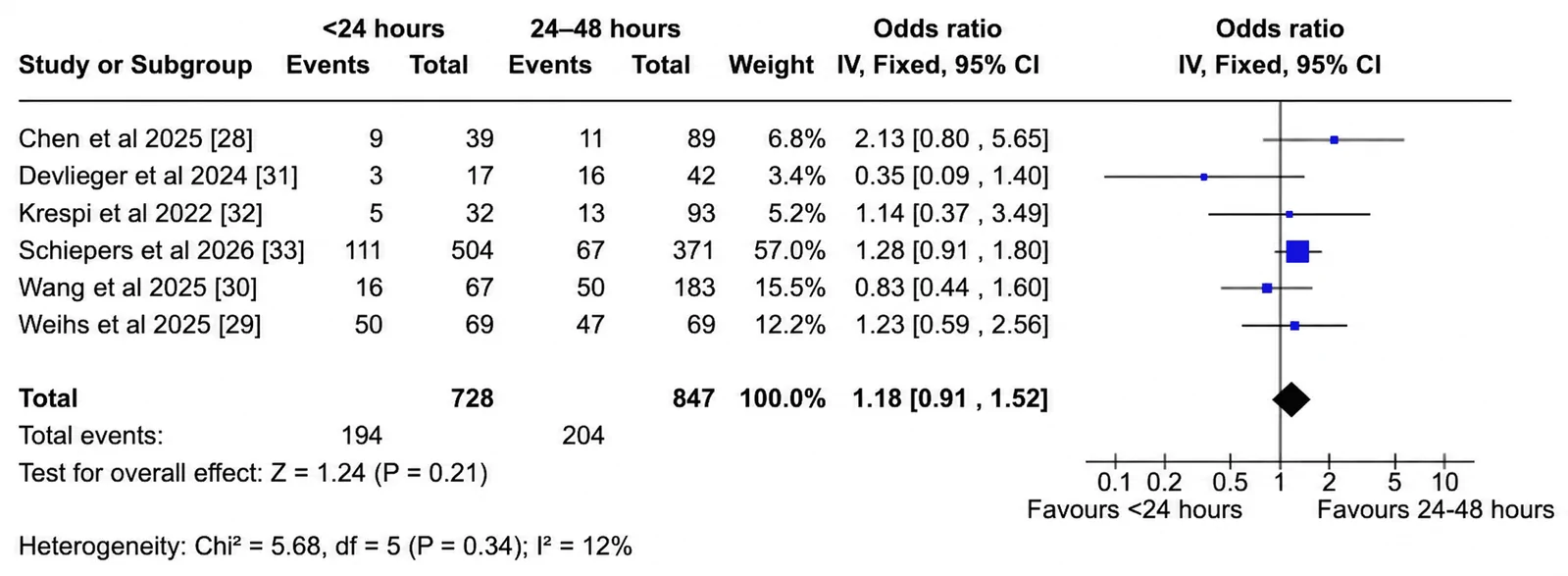
Forest plot comparing postoperative transfusion requirements between surgery performed within 24 hours and 24-48 hours after the last DOAC dose DOAC: direct oral anticoagulant; IV: inverse variance; CI: confidence interval; df: degrees of freedom [28-32]

Transfusion requirements: All six studies [28-33] reported postoperative blood transfusion requirements and were included in the meta-analysis. A total of 194 of 728 patients (26.6%) in the <24-hour group required a postoperative blood transfusion, compared with 204 of 847 patients (24.1%) in the 24-48-hour group. Pooled analysis demonstrated no statistically significant difference in transfusion requirements between the two groups (OR 1.18, 95% CI 0.91-1.52; p = 0.21) (Figure 3). Heterogeneity was low (I² = 12%).

Secondary Outcomes

**Figure 4:**
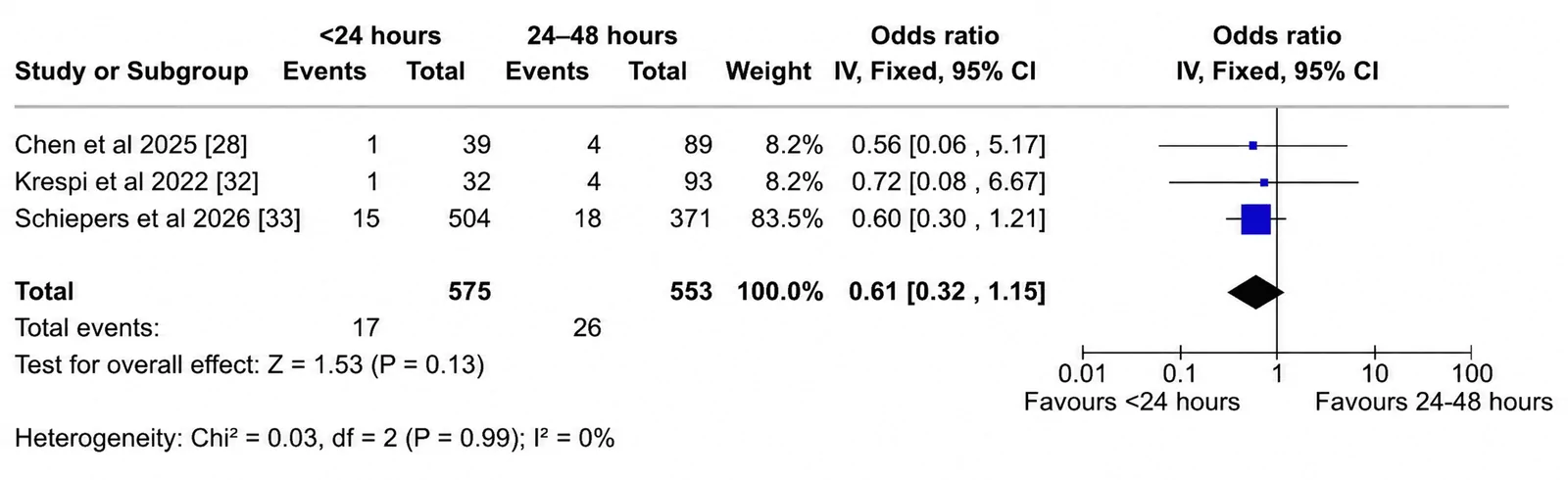
Forest plot comparing 30-day all-cause mortality between surgery performed within 24 hours and 24-48 hours after the last DOAC dose DOAC: direct oral anticoagulant; IV: inverse variance; CI: confidence interval; df: degrees of freedom [28,32,33]

30-day all-cause mortality: Three studies [28,32,33] reported 30-day all-cause mortality and were included in the meta-analysis. A total of 17 of 575 patients (3.0%) in the <24-hour group died within 30 days of surgery, compared with 26 of 553 patients (4.7%) in the 24-48-hour group. Pooled analysis demonstrated no statistically significant difference in 30-day mortality between the two groups (OR 0.61, 95% CI 0.32-1.15; p = 0.13) (Figure 4). No statistical heterogeneity was observed (I² = 0%).

**Figure 5:**
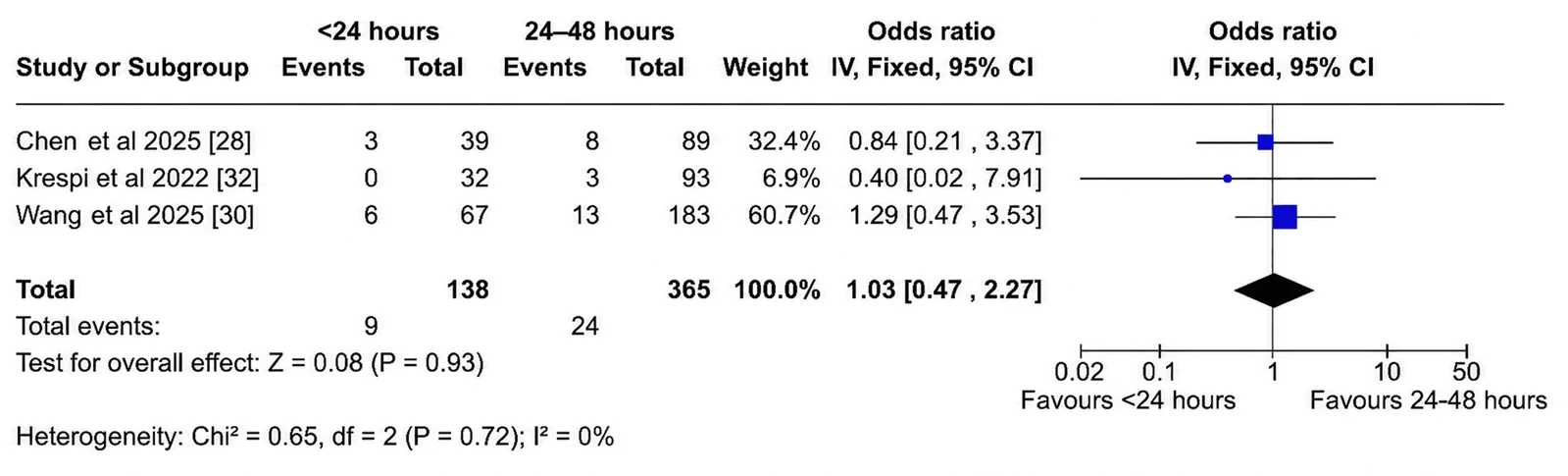
Forest plot comparing 90-day all-cause mortality between surgery performed within 24 hours and 24-48 hours after the last DOAC dose DOAC: direct oral anticoagulant; IV: inverse variance; CI: confidence interval; df: degrees of freedom [28,30,32]

90-day all-cause mortality: Three studies [28,30,32] reported 90-day all-cause mortality. A total of nine of 138 patients (6.5%) in the <24-hour group died within 90 days of surgery, compared with 24 of 365 patients (6.6%) in the 24-48-hour group. Pooled analysis demonstrated no statistically significant difference in 90-day mortality between the two groups (OR 1.03, 95% CI 0.47-2.27; p = 0.93) (Figure 5). No heterogeneity was observed (I² = 0%).

**Figure 6:**
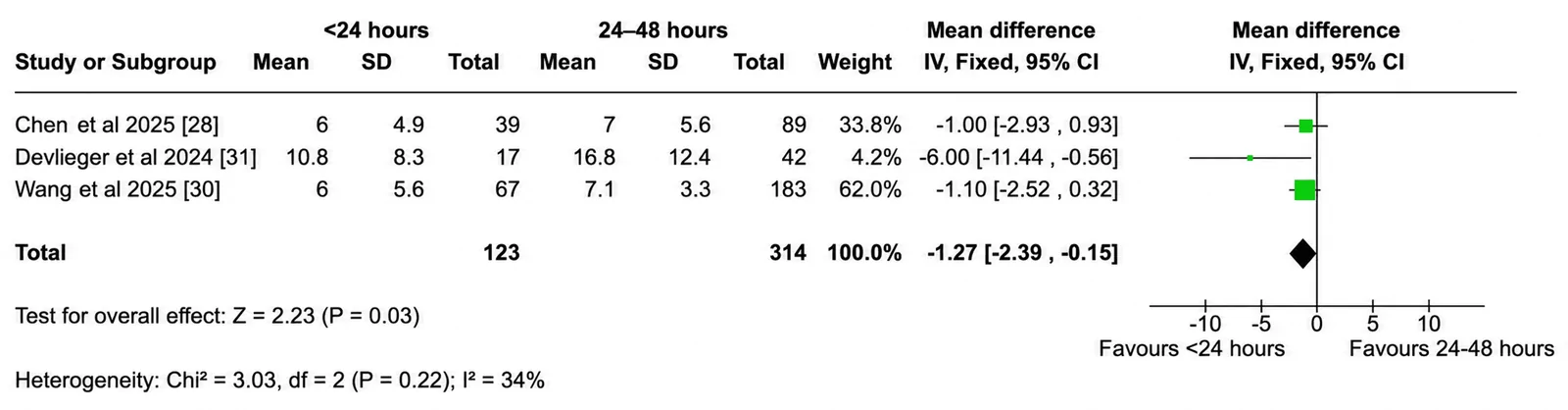
Forest plot comparing length of hospital stay between surgery performed within 24 hours and 24-48 hours after the last DOAC dose DOAC: direct oral anticoagulant; IV: inverse variance; CI: confidence interval; df: degrees of freedom [28,30,31]

Length of hospital stay: Three studies [28,30,31] reported length of hospital stay and were included in the meta-analysis. A statistically significant difference was observed between the two groups, with patients undergoing surgery within 24 hours experiencing a shorter hospital stay than those undergoing surgery between 24 and 48 hours. The pooled analysis demonstrated an MD of -1.27 days in favour of the <24-hour group (95% CI -2.39 to -0.15; p = 0.03) (Figure 6). Low-to-moderate heterogeneity was observed (I² = 34%).

Complications: Three studies [29-31] reported postoperative complications. Any one of the following complications was included as an event: haematoma, venous thromboembolism, pneumonia, acute kidney injury, myocardial infarction, and urinary tract infection. A total of 33 out of 153 patients sustained one of the pre-stated complications in the <24-hour group, while 112 out of 294 patients sustained a complication in the 24-48-hour group. Overall, complication rates were 21.6% and 38.1% in the <24-hour and 24-48-hour groups, respectively.

**Figure 7:**
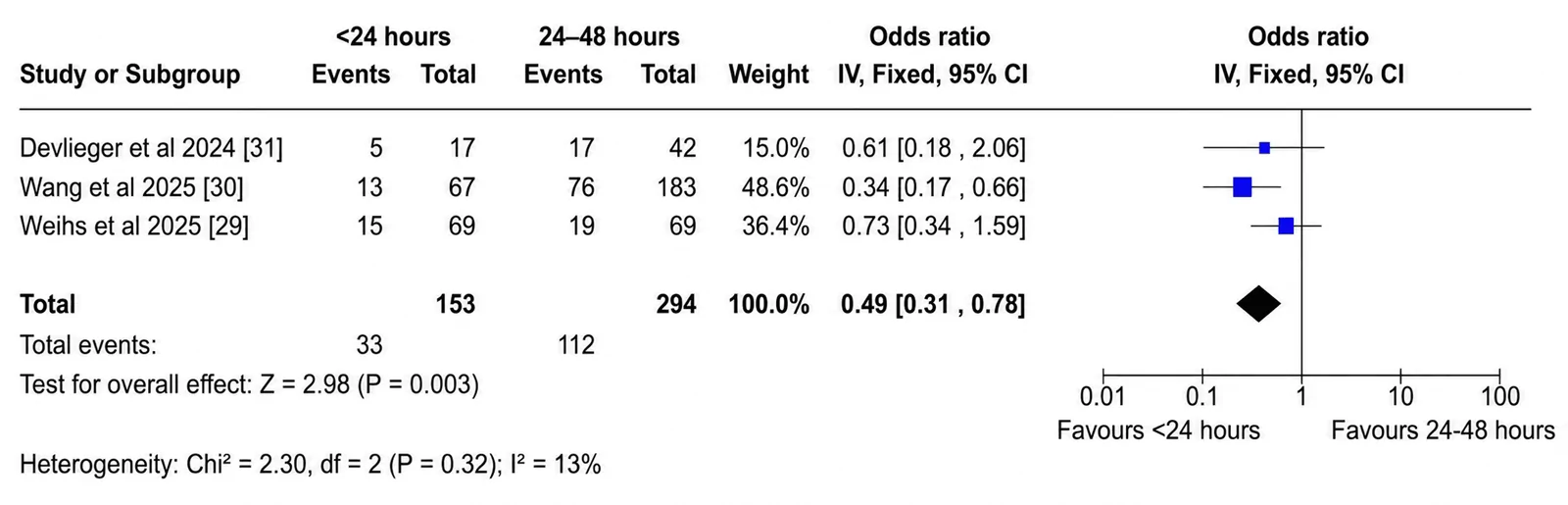
Forest plot comparing reported complications between surgery performed within 24 hours and 24-48 hours after the last DOAC dose DOAC: direct oral anticoagulant; IV: inverse variance; CI: confidence interval; df: degrees of freedom [29-31]

Meta-analysis demonstrated a statistically significant difference between the groups, with surgery performed within 24 hours being associated with a lower number of reported complications compared with surgery performed between 24 and 48 hours (OR 0.49, 95% CI 0.31-0.78; p = 0.003) (Figure 7). Low heterogeneity was observed (I² = 13%).

Operative time: Two studies reported operative duration. Chen et al. [28] and Schiepers et al. [33] found no significant difference in operative time between patients undergoing surgery within 24 hours and those undergoing surgery between 24 and 48 hours. In Chen et al., the mean operative time was 101.6 ± 61.4 minutes in the early surgery group and 102.4 ± 27.4 minutes in the delayed surgery group [28]. Similarly, Schiepers et al. [33] reported a median operative time of 96 minutes in both groups (IQR 77-119 vs 81-117), with a median difference of zero minutes (95% CI −4 to 4; p = 0.975), indicating no statistically significant difference in operative duration between the groups.

Risk of Bias Assessment

**Figure 8:**
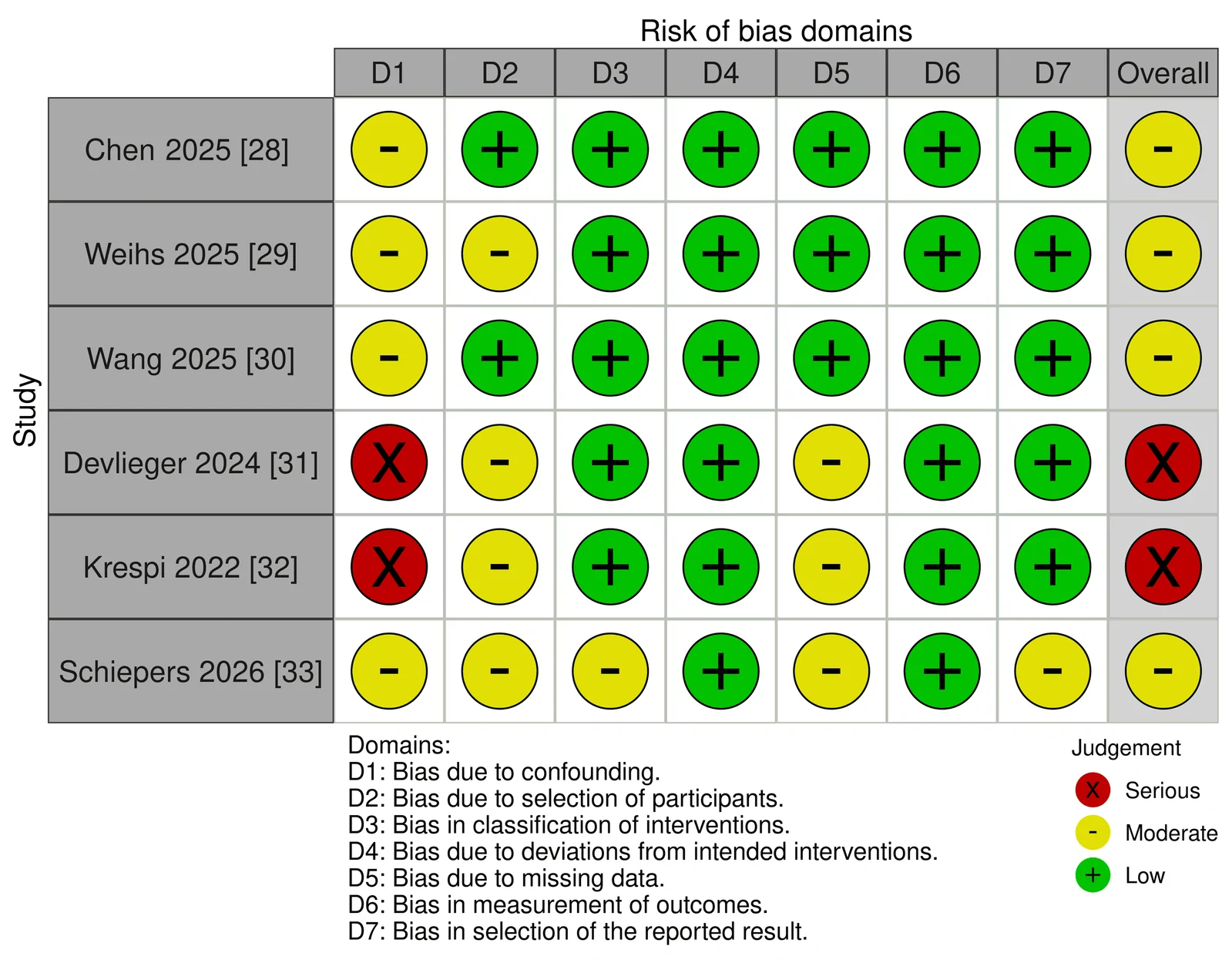
Risk of bias analysis using ROBVIS tool ROBVIS: Risk-of-Bias Visualisation [28-33]

Risk of bias was assessed using the ROBINS-I tool. Devlieger et al. [31] and Krespi et al. [32] were judged to be at serious overall risk of bias, whereas the remaining four studies [28-30,33] were assessed as having a moderate risk of bias. The principal source of bias across the included studies was confounding, reflecting the non-randomised allocation of surgical timing and the potential for confounding by indication, whereby patients undergoing delayed surgery may have differed systematically from those treated earlier. In contrast, the risk of bias arising from deviations from intended interventions, outcome measurement, and selective reporting was generally low across all the included studies, largely owing to the objective nature of the reported outcomes. Detailed domain-level assessment is presented in Figure 8.

Discussion

In this systematic review and meta-analysis, hip fracture surgery performed within 24 hours of the last DOAC dose was compared with surgery undertaken between 24 and 48 hours after the last DOAC dose. Although delayed surgery was associated with a statistically significant reduction in perioperative haemoglobin concentration difference, the absolute difference was small (MD 0.29 g/dL) and is unlikely to be clinically meaningful. This interpretation is supported by the finding that postoperative transfusion requirements did not differ significantly between the two groups. Collectively, these findings suggest that delaying surgery confers little clinically meaningful benefit with regard to perioperative blood loss.

With respect to all-cause mortality, pooled analysis demonstrated no significant difference in either 30- or 90-day mortality between patients undergoing surgery within 24 hours and those treated between 24 and 48 hours. These findings suggest that expedited operative intervention within 24 hours achieves comparable short-term mortality outcomes to delayed surgery without an apparent increase in mortality risk.

Length of hospital stay was significantly shorter among patients undergoing surgery within 24 hours of the last DOAC dose (MD -1.27 days). This finding is consistent with previous studies in the general hip fracture population, including that of Simonovic et al. [34] who demonstrated that earlier operative intervention was independently associated with a shorter hospital stay in both adjusted and unadjusted analyses. Given the substantial healthcare burden associated with hip fractures, even a modest reduction in length of stay may improve patient flow, reduce healthcare costs, and optimise hospital resource utilisation [35].

Postoperative complications were significantly less frequent among patients undergoing surgery within the early group. Again, this finding is consistent with the wider hip fracture literature, where delayed surgery has been associated with higher rates of medical complications, including respiratory infections, delirium, and venous thromboembolism [36]. Although the studies included in this review were observational and cannot establish causality, the consistency of these findings suggests that the correlation of early surgery and a reduction in complications is also seen in patients receiving DOAC therapy.

Operative duration was comparable between the early and delayed surgery groups in the two studies that reported this outcome [28,33]. In theory, increased intraoperative bleeding in patients with recent DOAC exposure could make surgery more challenging by obscuring the operative field and requiring additional time to achieve haemostasis. One might therefore expect surgery performed within 24 hours to be associated with longer operative times; however, this was not observed in the available studies. Nevertheless, operative duration should be interpreted with caution as a surrogate marker of surgical difficulty. Many hip fracture fixation procedures are performed using standardised, fluoroscopy-guided techniques and limited surgical exposure, where a bloodless field is not always required [37]. Therefore, even if intraoperative bleeding is increased, it may not necessarily translate into a longer operative time or complexity.

Limitations

This systematic review and meta-analysis has several limitations. Firstly, the literature search was restricted to PubMed, Embase, and the Cochrane Library; consequently, relevant studies indexed in other databases or unpublished sources may have been missed. Furthermore, the available evidence base remains limited. As DOACs have only been widely used since the late 2000s [38], relatively few studies have specifically evaluated the impact of surgical timing in anticoagulated hip fracture patients.

Secondly, pooled estimates were disproportionately influenced by larger studies, particularly Schiepers et al. [33], which contributed more than half of the statistical weighting in several analyses. Consequently, the overall findings may have been driven largely by a single study, while the remaining studies included comparatively small patient cohorts.

Thirdly, clinical heterogeneity existed with respect to the anticoagulant agents studied. Although collectively referred to as DOACs, these medications possess distinct pharmacokinetic and pharmacodynamic properties, including differences in half-life, clearance, and mechanism of action. For example, Wang et al. [30] excluded dabigatran-treated patients, whereas Chen et al. [28] reported a predominance of apixaban users. Such variation may limit the generalisability of pooled estimates and could have influenced observed outcomes.

Finally, the overall quality of evidence was limited by study design. With the exception of Weihs et al. [29], all included studies were retrospective observational cohorts, and no randomised controlled trials were identified. Consequently, the findings remain susceptible to selection bias and confounding by indication, whereby patients undergoing delayed surgery may have differed systematically from those treated earlier. Although most studies adjusted for important baseline characteristics, the observational nature of the evidence precludes definitive conclusions regarding causality.

## Conclusions

The findings of this systematic review and meta-analysis suggest that delaying hip fracture surgery due to DOAC therapy offers little clinically meaningful benefit. Although delayed surgery was associated with a small reduction in perioperative haemoglobin loss, this did not translate into lower transfusion requirements or improved survival. Conversely, surgery within 24 hours was associated with a shorter length of hospital stay and fewer postoperative complications, without evidence of increased mortality or operative duration. Collectively, these findings support early surgery within 24 hours of the last DOAC dose in appropriately selected patients and indicate that DOAC use alone should not routinely justify delaying hip fracture surgery.
